# Peripheral endocannabinoid concentrations are not associated with verbal memory impairment during MDMA intoxication

**DOI:** 10.1007/s00213-017-4787-2

**Published:** 2017-11-16

**Authors:** E. Haijen, M. Farre, R. de la Torre, A. Pastor, E. Olesti, N. Pizarro, J. G. Ramaekers, K. P. C. Kuypers

**Affiliations:** 1 Department of Neuropsychology and Psychopharmacology, Faculty of Psychology and Neuroscience, Maastricht University, Maastricht, The Netherlands; 2 Integrative Pharmacology & Neurosciences Systems Research Group, Institut Hospital del Mar d’Investigacions Mèdiques, Barcelona, Spain; 3 Universitat Autonoma de Barcelona, Barcelona, Spain; 4 Clinical Pharmacology, Hospital Universitari Germans Trias i Pujol, Badalona, Spain; 5 Spanish Biomedical Research Centre in Physiopathology of Obesity and Nutrition (CIBEROBN), Santiago de Compostela, Spain; 6 Universitat Pompeu Fabra, CEXS-UPF, Barcelona, Spain

**Keywords:** MDMA, Verbal memory, 5-HT2 receptor, Ketanserin, Endocannabinoids, 2-AG, AEA

## Abstract

**Background:**

Preclinical data have suggested involvement of the endocannabinoid (eCB) system in MDMA-induced memory impairment. Clinical research has shown that blockade of the 5-HT_2_ receptor nulls memory impairment during MDMA intoxication. Interestingly, studies have demonstrated that the eCB and the 5-HT system interact. It was hypothesized that MDMA would cause an increase in eCB concentrations together with a decrease in memory performance, and that combining MDMA with a 5-HT_2_ receptor blocker ketanserin would lead to a counteraction of the MDMA effects on eCB concentrations and memory.

**Methods:**

Twenty healthy recreational polydrug users entered a double-blind placebo-controlled within-subject study. Participants received a pre-treatment (ketanserin 40 mg, placebo) followed 30 min later by a treatment (MDMA 75 mg, placebo). Verbal memory was tested by means of a 30-word learning test. Endocannabinoid concentrations (anandamide (2-AG); *N*-arachidonylethanolamine (AEA)) were assessed in blood at baseline, before (90 min post-treatment) and after cognitive tests (150 min post-treatment).

**Results:**

Findings showed that MDMA impaired memory 90 min post-treatment in the word learning task. This effect was a replication of previous studies using the same dose of MDMA (75 mg) and the same learning paradigm. Contrary to our hypothesis, MDMA did not affect eCB concentrations, nor did ketanserin block MDMA-induced memory impairment. Ketanserin caused an increase in AEA concentrations, 180 min after administration.

**Conclusion:**

Current findings suggest that peripherally measured endocannabinoids are not associated with the verbal memory deficit during MDMA intoxication. Trial registration number: NTR3691.

## Introduction

Previous placebo-controlled experimental studies have consistently shown that a single dose (75 mg) of (R,S)-3,4 methylenedioxymethamphetamine (MDMA) impairs memory for verbal information (e.g., de Sousa Fernandes Perna et al. 2014; Kuypers and Ramaekers 2005). The neurobiological mechanism underlying this impairment has been studied and it was suggested that the MDMA-induced elevation in plasma cortisol concentrations was not related to the observed deficit (Kuypers et al. 2013). van Wel and colleagues demonstrated that blockade of the serotonin-2A (5-HT_2A_) receptor, by means of a single dose of ketanserin, prevented the memory impairment after a single dose of MDMA (van Wel et al. 2011). The detailed neurobiological mechanism behind the MDMA-induced memory deficit has yet to be elucidated.

There is evidence suggesting that the serotonergic neurotransmission system is modulated by the endocannabinoid (eCB) system. Specifically, the CB_1_ receptor modulates the excitability of dorsal raphe serotonin neurons (Haj-Dahmane and Shen 2011), and more relevant in the context of the findings by van Wel et al. (2011), 5-HT_2A_ receptor activation stimulates the formation and release of 2-arachidonoylglycerol (2-AG), an endocannabinoid (Parrish and Nichols 2006). The best characterized eCBs are 2-AG and AEA (*N*-arachidonylethanolamine, anandamide), and both of these lipids exert agonist activity at CB_1_ and CB_2_ receptors. They are synthetized on an “on-demand” basis and crucial in certain forms of neuronal plasticity (Curran et al. 2016). Since MDMA acts on the 5-HT_2A_ receptor (Erritzoe et al. 2011; van Wel et al. 2011), it can be hypothesized that this also has implications for endocannabinoid concentrations.

Converging data suggest that MDMA and (exogenous and endogenous) cannabinoids interact pharmacologically (Valverde and Rodriguez-Arias 2013). Both MDMA-induced conditioned place preference and self-administration in rats are under endogenous tonic control by the endocannabinoid system (Braida et al. 2005; Braida and Sala 2002). Low doses of THC have been demonstrated to modulate MDMA-induced behavioral effects, decreasing conditioned place preference (Robledo et al. 2007). In line with this, combined THC-MDMA administration led to synergistic effects on working memory in rats; MDMA in low-to-high doses led to an exacerbation of the THC-induced memory impairment (Young et al. 2005). Interestingly, MDMA-induced memory impairment in rats could be blocked with a CB_1_ receptor antagonist (Nawata et al. 2010).

Together, these data suggest involvement of the endocannabinoid system in MDMA-induced memory impairment. The present study was therefore set up to study the association between endocannabinoid concentrations and verbal memory performance during MDMA intoxication. It was hypothesized that MDMA would cause an increase in eCB concentrations together with a decrease in verbal memory performance. In addition, given the role of the 5-HT_2A_ receptor in memory, and its interaction with the eCB system, it was hypothesized that pretreatment with ketanserin, a 5-HT_2A_ receptor blocker, would counteract the effects of MDMA on endocannabinoid release and memory.

## Methods

### Participants

Participants were 20 healthy polydrug MDMA users (mean (SD) age = 21.2 (2.6); 8 females), who previously used ecstasy/MDMA (16.8 (23.2) times) and other recreational drugs (e.g., cocaine, amphetamine, and cannabis). The mean (SD) verbal IQ was 103.9 (4.9) as determined by the National Adult Reading Test on the training session, preceding the test sessions (Bright et al. 2002).

Participants were recruited through advertisements in university buildings, via a website (digi-prik.nl), and by word of mouth.

### Design and treatments

The study was conducted according to a two-by-two double-blind, placebo-controlled within-subject design with pre-treatment (ketanserin 40 mg or placebo) preceding the treatment (MDMA 75 mg or placebo) by 30 min (Brogden and Sorkin 1990; Sharpley et al. 1994). A double-dummy procedure was used to control for differences in *T*_max_ between both drugs. *T*_max_ of MDMA is 2 h (de la Torre et al. 2004) and *T*_max_ of ketanserin is between 0.5 and 4 h (Heykants et al. 1986; Persson et al. 1991; Reimann et al. 1983). The timing of the (pre-) treatment was based on similar research conducted by the same group (van Wel et al. 2011; van Wel et al. 2012) where it was shown that the MDMA-induced elevated mood state was blocked by ketanserin (van Wel et al. 2012).

The 75-mg dose of MDMA was selected because it has consistently been shown to impair memory performance and to produce robust subjective mood changes in a number of previous studies from our lab (Kuypers and Ramaekers 2005, 2007; Kuypers et al. 2016; Ramaekers et al. 2009). Ketanserin 40 mg represents a regular therapeutic dose that blocks 91% of 5-HT_2_ receptors (Brogden and Sorkin 1990; Sharpley et al. 1994).

A permit for obtaining, storing, and administering MDMA was obtained from the Dutch drug enforcement administration. Randomization of pre-treatment and treatment conditions was generated by means of a Latin square, with each participant being assigned to a treatment sequence.

### Procedures

Prior to participation, all participants were medically assessed by a physician, who examined general health, including an ECG, and who took blood and urine samples for standard chemistry and hematology. In addition, participants were familiarized with the procedures, tests, and questionnaires on a training day, preceding actual test days. On the training day, participants were shown the questionnaires so that they knew what they looked like, and they had to run through all tests so that they understood what was expected. The order of the questionnaires and tests was kept the same as on an actual test day. No learning effects were expected on the tests that were used though parallel versions of the word learning test were used so that each test day participants had to learn a new list and no interference was possible.

Participants were requested to abstain from any drug use 1 week before the medical examination until the last test day. They were asked not to use any caffeinated or alcoholic beverages 24 h before testing and to get a normal night sleep as assessed with the Groninger Sleep Scale.

A test day started at 9 am with a screen for drugs of abuse in urine (THC/opiates/cocaine/amphetamines/methamphetamines), a breathalyzer ethanol test, and a pregnancy test for women. When tests were negative, participants had breakfast and a blood sample was taken. At 9:30 am, participants received the pre-treatment followed 30 min later by the treatment. Participants were then seated in a waiting room. At 11:25 am, a second blood sample was taken. Thereafter, the word learning task was assessed followed by a 1-h test battery consisting of social behavior tests (Approach Avoidance Test with emotional and social situation stimuli, Processing of Sounds Task) and questionnaires (Dissociative Experiences Scale, Clinician Administered Dissociative States Scale, Profile of Mood States); these data are reported elsewhere (Kuypers et al. 2017; Puxty et al. 2017). The test day ended with the collection of a third blood sample. The study consisted of four test days that were minimally separated by a 7-day wash-out period.

The study was performed in accordance with the Helsinki Declaration of 1975, and its subsequent amendments, and was approved by the Medical Ethics Committee of the Academic Hospital of Maastricht and the University of Maastricht. All participants gave their written informed consent after description of the study and they were paid upon completion of the testing periods for their participation.

### Word learning task

The 30-word learning task consists of 30 Dutch mono-syllabic meaningful nouns (*N* = 18) and adjectives (*N* = 12) which are consecutively presented on a computer screen (Kuypers et al. 2013; Rey 1958). The words are either neutral (*N* = 6) or had a valence (positive (*N* = 12) or negative (*N* = 12)). The words in the lists of the five parallel versions (4 + 1 for test days + training session) had been matched for abstraction. Participants had to recall verbally as many words as possible (immediate recall). This procedure was repeated three times; immediate scores were summed to comprise the “total immediate recall” score. After a 30-min delay, participants were asked to recall (“delayed recall”) as many of the previously learnt words as possible. Hereafter, participants were given a delayed recognition task containing 30 new words and all the words of the previously shown list. Participants’ task was to indicate whether the presented word was a new one or one from the original list. Dependent variables were number of correct recalled words per trial, the immediate recall (IR) total score, the delayed recall (DR) score, the delayed recognition score (total number of correct items of the original list; max score = 30), and corresponding RTs.

### National Adult Reading Test

The Dutch version of the National Adult Reading Test was used to estimate the premorbid verbal intelligence of participants (Bright et al. 2002; Schmand et al. 1991).

### Groninger Sleep Scale

The Groninger Sleep Scale assesses sleep quality and quantity (hours of sleep). It consists of 15 dichotomous questions about sleep complaints and an open question concerning the duration of sleep. The number of hours sleep and the total score on this questionnaire were compared over the four test days to ascertain that participants had an equal amount of sleep quantity and quality before each test day (Mulder-Hajonides van der Meulen et al. 1980).

### Pharmacokinetics and endocannabinoid concentrations

Blood samples preserved with EDTA were collected three times on each test day, at baseline, 90 min after treatment, and 150 min after treatment, in order to determine endocannabinoid (AEA, 2-AG) concentrations and pharmacokinetics of MDMA and ketanserin. Samples were centrifuged immediately and resulting plasma was stored at − 20 °C until analysis.

#### MDMA and ketanserin blood concentrations

MDMA was determined by gas chromatography coupled to mass spectrometry using a method previously described by Pizarro et al. (2002). Ketanserin was determined by liquid chromatography coupled to mass spectrometry. Samples (200 μL of plasma) were purified with Ostro Pass-through Sample Preparation Plates (Waters, MA, USA) and 600 μL of acetonitrile with 0.1% formic acid was used as the elution solvent. After mixing, vacuum was applied and the collected mixture was evaporated to dryness at 15 psi and 40 °C. Extract was reconstituted with 100 μL of ammonium formate 0.02% at pH 5 and acetonitrile (50:50 *v*/*v*). Quantification was performed in a HPLC system coupled to a triple-quadrupole (6410 Triple Quad LC-MS; Agilent) mass spectrometer with an electrospray interface. The chromatographic separation was done using a C18 column (Kinetex, 100 mm × 3 mm × 1.7 μm, Phenomenex, CA, USA). The mobile phase was ammonium formate 0.02% at pH 5 and acetonitrile in an isocratic mode (50:50 *v*/*v*) at a flow rate of 0.45 mL/min. All compounds were monitored in positive ionization using the multiple reaction mode mass/charge (*M* + 1/*z*). Parameters for the identification of analytes were as follows: ketanserin 396 → 146, 189, 208; fragmentor (F) 200 V, collision energy (CE) 15 V; and pirenperone 394 → 119, 159, 187, F200, CE15.

#### Endocannabinoid concentrations

The analysis of the endocannabinoids AEA and 2-AG in plasma was performed by a validated method previously described (Pastor et al. 2014). Briefly, aliquots of 0.5 mL of plasma were transferred to 12-mL glass tubes, spiked with deuterated internal standards, diluted with 0.1 M ammonium acetate buffer (pH 4.0), and extracted with tert-butyl methyl ether. The dry organic extracts were reconstituted in 100 μL of a mixture water:acetonitrile (10:90, *v*/*v*) with 0.1% formic acid (*v*/*v*) and transferred to HPLC vials. Twenty microliters was injected into the LC/MS-MS system. An Agilent 6410 triple quadrupole (Agilent Technologies, Wilmington, DE, USA) equipped with a 1200 series binary pump, a column oven, and a cooled auto-sampler (4 °C) was used. Chromatographic separation was carried out with a ACQUITY UPLC C18-CSH column (3.1 × 100 mm, 1.8 μm particle size) (Waters, Yvelines Cedex, France) maintained at 40 °C with a mobile phase flow rate of 0.4 mL/min. The composition of the mobile phase was as follows: A: 0.1% (*v*/*v*) formic acid in water; B: 0.1% (*v*/*v*) formic acid in acetonitrile. Detection was done by selection reaction monitoring (SRM). Quantification was performed by isotope dilution. Deuterated internal standards were obtained from Cayman Chemical (Ann Arbor, MI, USA), and solvents were from Merck (Darmstadt, Germany).

### Statistical analysis

Data of the word learning task (WLT) and the concentrations of eCBs entered a general linear model (GLM) repeated measures ANOVA (SPSS, version 24.0) with pre-treatment (two levels: ketanserin, placebo) and treatment (two levels: MDMA, placebo) as main within-subject factors. IR trial (three levels) was included as extra within-subject factor for the WLT. Data of the Groninger Sleep Scale (sleep quantity and sleep quality) entered repeated measures ANOVA with test day (four levels) as within-subject factor to test whether participants had an equal amount of sleep quantity and quality before each test day.

Paired sample *t* tests were conducted to investigate the difference in MDMA and ketanserin concentrations in conditions where MDMA or ketanserin was administered alone and in combination.

The alpha criterion level of statistical significance for all analyses was set at *p* = 0.05. Partial eta squared (partial *ƞ* ^*2*^) is reported in case of significant effects to demonstrate the effect’s magnitude, where 0.01 is defined as small, 0.06 as moderate, and 0.14 as large. Partial eta squared is based on Cohen’s *f* which defines small, medium, and large as, respectively, 0.10, 0.25, and 0.50 which corresponds to *η* ^2^ of 0.0099, 0.0588, and 0.1379 (Richardson 2011).

## Results

### Word learning task

**Fig. 1:**
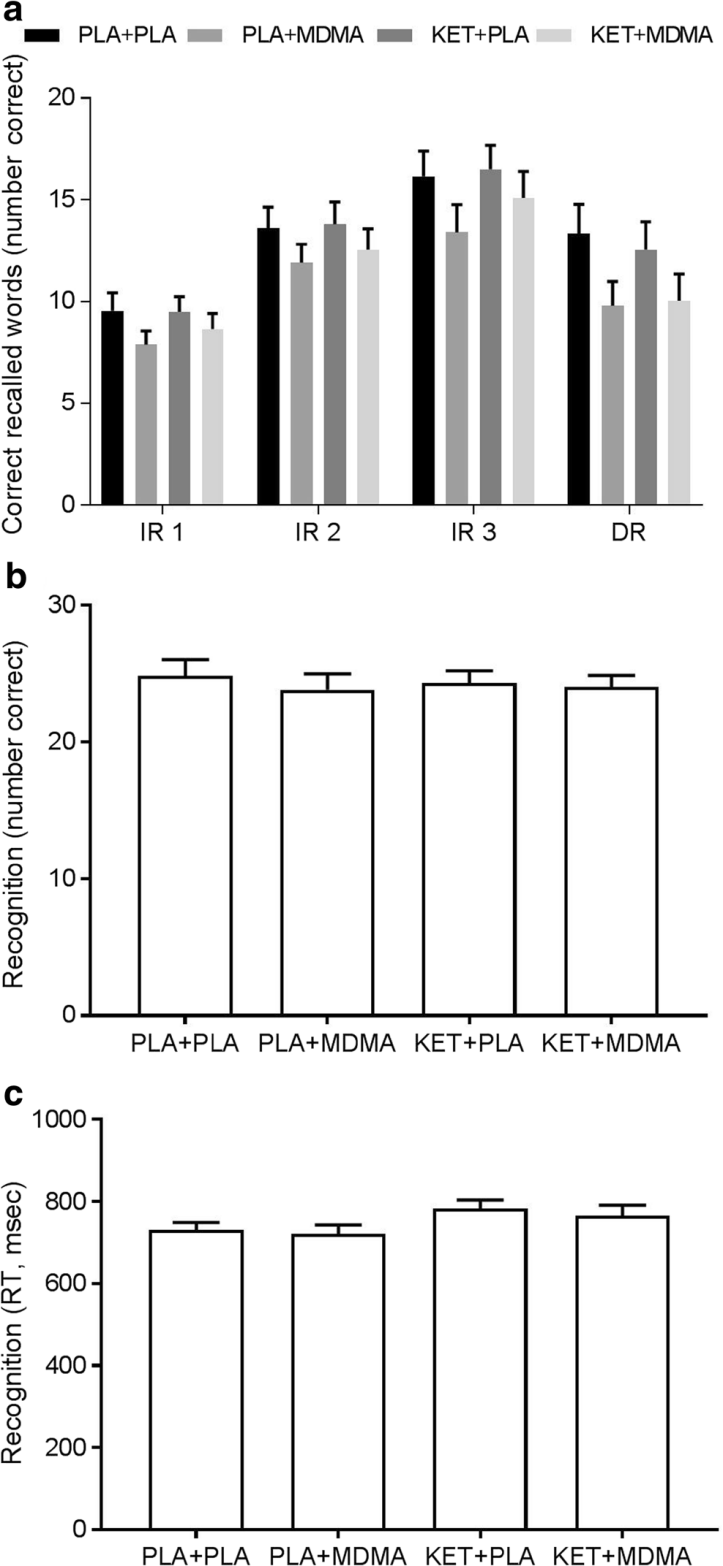
Mean (SE) of number correct recalled words (**a**), number of correct recognized words (**b**), and corresponding reaction times (**c**) in the word learning task per treatment condition. PLA placebo, KET ketanserin

GLM RM ANOVA analysis revealed a main effect of treatment on immediate recall total (*F*_1, 19_ = 5.48, *p* = 0.03, partial *ƞ* ^*2*^ = 0.22). There was also a main effect of trial on immediate recall (IR) per trial indicating that performance increased over trials (*F*_1, 19_ = 91.02, *p* < 0.001, partial *ƞ* ^*2*^ = 0.83). Under influence of MDMA, participants recalled on average 1.6 words less per trial, and in total 4.8 words less, compared to placebo. Analysis also revealed a main effect of treatment (*F*_1, 19_ = 8.76, *p* = 0.008, partial *ƞ* ^*2*^ = 0.32) on delayed recall (DR). Participants recalled on average 3 words less, 30 min after the initial learning phase, compared to placebo. There was no main effect of pre-treatment or valence, or an interaction effect between factors. There was no main effect of pre-treatment or an interaction effect of pre-treatment by treatment on IR trial, IR total, or DR (Fig. 1a).

Analysis revealed no statistically significant main effect of treatment, pre-treatment, or their interaction on number of correct recognized words (Fig. 1b). A main effect of pre-treatment (*F*_1, 19_ = 5.94, *p* = 0.02, partial *ƞ* ^*2*^ = 0.24) was found on reaction time related to correct recognized words. Participants were on average 49 ms slower under influence of ketanserin compared to placebo (Fig. 1c). There was no main effect of treatment or pre-treatment by treatment interaction on reaction time in the recognition task.

### Groninger Sleep Scale

Analysis of the Groninger Sleep Scale, which served as a control measure, showed no difference in sleep quality (*F*_3, 57_ = 2.11, *p* = 0.11, partial *ƞ* ^*2*^ = 0.10) and quantity (*F*_3, 57_ = 0.35, *p* = 0.79, partial *ƞ* ^*2*^ = 0.02) between the four test days. Participants slept on average 6 h and 59 min (SD = 0.9) on the night prior to a test day, and they had and average sleep quality score of 2.5 (SD = 2.3).

### Pharmacokinetics and endocannabinoid concentrations

#### Endocannabinoid concentrations

**Fig. 2:**
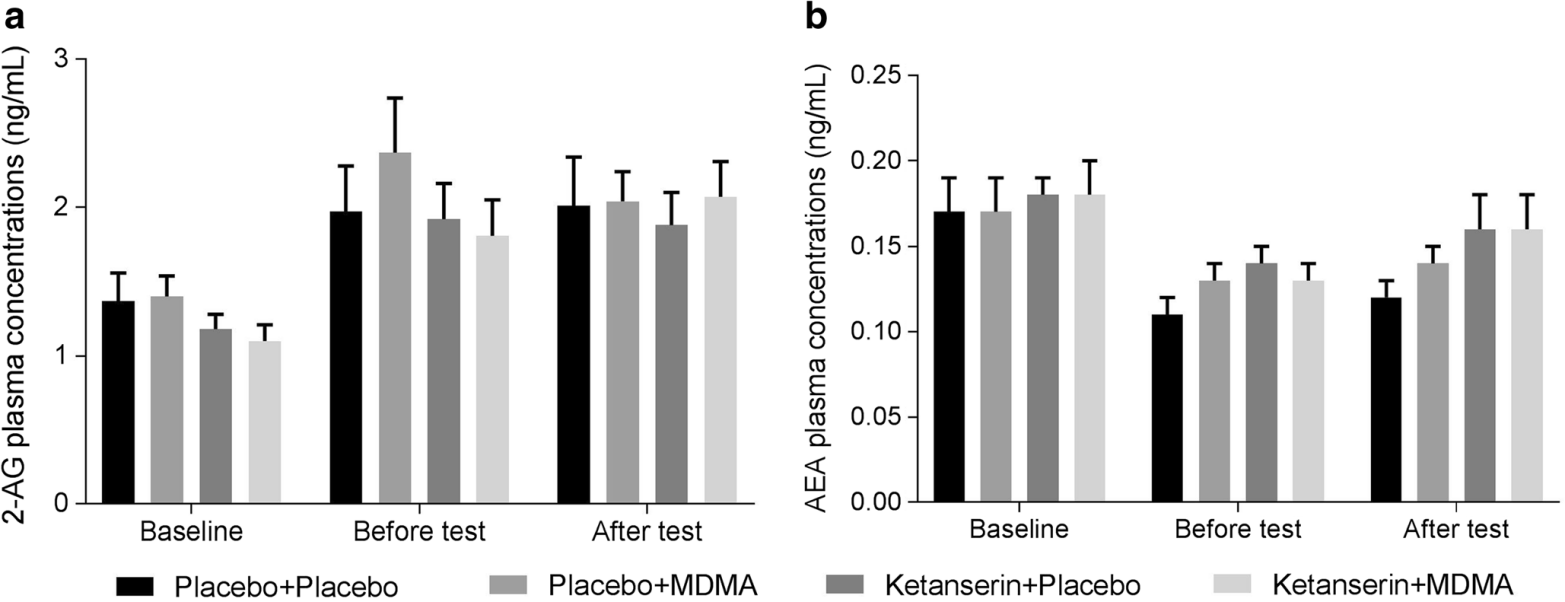
Mean (± SE) plasma concentrations of 2-AG (**a**) and AEA (**b**) in the four treatment conditions, and at baseline, before tests (90 min after treatment, respectively, 120 min after pre-treatment), and after tests (150 min after treatment, respectively, 180 min after pre-treatment)

Analyses revealed a main effect of pre-treatment (*F*_1, 11_ = 10.6; *p* = 0.005; partial *ƞ* ^*2*^ = 0.41) on plasma AEA concentrations (ng/mL) assessed after the tests. AEA concentrations were higher 180 min after ketanserin administration compared to placebo. There were no differences in endocannabinoid (2-AG, AEA) plasma concentrations at baseline and there were no other main or interaction effects on 2-AG or AEA concentrations (Fig. 2).

Since visual inspection of the data suggested differences between baseline concentrations of 2-AG and AEA, and the two subsequent measurements, before and after the cognitive tests, an extra explorative GLM was run to test whether endocannabinoid concentrations varied naturally over time. The extra GLM demonstrated a main effect of “measurement” on 2-AG (*F*_2, 30_ = 15.90; *p* < 0.001; partial *ƞ* ^*2*^ = 0.51) and AEA concentrations (*F*_2, 30_ = 13.90; *p* < 0.001; partial *ƞ* ^*2*^ = 0.47). Pairwise comparisons revealed that 2-AG baseline concentrations were significantly lower compared to measure 2 (before cognitive tests) and measure 3 (after cognitive tests); 2-AG concentrations did not statistically differ between measurements 2 and 3. Baseline AEA concentrations were significantly lower compared to the second measurement while there were no differences between baseline concentrations and the third measure or between the second and the third measure.

#### MDMA and ketanserin blood concentrations

Paired sample *t* tests showed that MDMA plasma concentrations (ng/mL) did not statistically differ between the MDMA alone condition (mean (± SE): 90 min post-MDMA: 134.8 (16.6); 150 min post-MDMA: 186.0 (17.7)) and the condition where MDMA was combined with ketanserin (mean (± SE): 90 min post-MDMA: 126.7 (15.1); 150 min post-MDMA: 182.9 (14.7)). The same was shown for ketanserin plasma concentrations (ng/mL) that did not differ between the ketanserin alone condition (mean (± SE): 90 min post-MDMA: 54.9 (7.6); 150 min post-MDMA: 64.5 (6.0)) and the condition where ketanserin was combined with MDMA (mean (± SE): 90 min post-MDMA: 59.0 (8.8); 150 min post-MDMA: 61.5 (5.4)).

## Discussion

The aim of the present study was to investigate the neurobiological mechanism underlying memory impairment during MDMA intoxication. Based on previous research, it was hypothesized that MDMA would cause an increase in eCB concentrations together with a decrease in memory performance. In addition, it was hypothesized that the combination of MDMA and a 5-HT_2A_ receptor blocker, ketanserin, would counteract this endocannabinoid release and the memory deficit. Findings showed that MDMA caused memory impairment in the verbal word learning task. This effect was a replication of previous studies using the same dose of MDMA (75 mg) and the same learning paradigm (Kuypers et al. 2016; Kuypers and Ramaekers 2005). Contrary to our hypothesis, MDMA did not affect eCB concentrations nor did ketanserin block the MDMA-induced memory impairment. Ketanserin caused an increase in AEA concentrations, 180 min after administration.

While preclinical studies have shown an elevation in eCB concentrations (Braida et al. 2005; Braida and Sala 2002), the present study did not demonstrate a significant increase in plasma eCB concentrations in healthy recreational drug users after a single dose of MDMA. This absence of MDMA effects on eCB concentrations could potentially be attributed to the difference in dosing and dosing schemes with “extreme” dose and dosing schemes in preclinical research exceeding “normal” dose ranges in humans (Easton and Marsden 2006; Green et al. 2012). In the study of Nawata and colleagues for example, endocannabinoid concentrations were elevated in mice after treatment with MDMA for seven subsequent days. The resulting memory impairment was reversed by a CB_1_ antagonist (Nawata et al. 2010). The MDMA-induced memory impairment which has consistently been demonstrated in human placebo-controlled studies seems to be unrelated to the endocannabinoid system.

The fact that endocannabinoid concentrations in the present study were assessed at the peripheral level, in blood plasma, could be another potential explanation for the absence of MDMA effects on eCB concentrations. It is possible that peripheral eCB concentrations might not reflect central concentrations accurately and preclinical work has previously shown that AG and AEA concentrations measured at the peripheral (plasma) and central level (cerebrospinal fluid (CSF)) did not correlate significantly (Jumpertz et al. 2011). While assessing biological parameters at the central level by drawing CSF is invasive, it is very relevant to conduct these measures and to compare central and peripheral markers in a placebo-controlled MDMA study prior to conducting behavioral studies. This information could fine-tune and optimize the timing and scheduling of tests.

While an MDMA effect on eCB concentrations was absent, data of the 2-AG and AEA concentrations, measured at three different time points, suggested time-related differences in concentrations. This motivated an extra statistical analysis including the three time points (9:00 am, 11:30 am, and 12:30 pm). It was shown that 2-AG concentrations increased during the test day and relative to baseline, and that AEA concentrations decreased. Interestingly, preclinical work has shown that endocannabinoids have a diurnal release pattern which is opposite to 2-AG and AEA, with higher AEA concentrations in a selection of brain areas (nucleus accumbens, prefrontal cortex, striatum, and hippocampus) in the dark or “active” phase and high concentrations for 2-AG during the light or “resting” phase (Valenti et al. 2004). Although this pattern in rats is opposite to our findings, the fact that eCBs have a diurnal rhythm is interesting. Human research has also shown that eCBs have a specific rhythm and our data are in line with this, with continuously increasing 2-AG concentrations across the morning, peaking in the early- to mid-afternoon, and decreasing AEA concentrations during the day (Hanlon et al. 2015; Vaughn et al. 2010).

Preclinical studies have in addition also shown that eCB concentrations do not only fluctuate over time but display different release patterns in different brain locations, showing for example higher AEA concentrations during resting phases in CSF and hypothalamus, while these concentrations are low in other brain structures like the hippocampus and the prefrontal cortex, both known to play a role in memory (Murillo-Rodriguez et al. 2006). In addition, preclinical research has demonstrated that AEA plays a central role in memory consolidation, while 2-AG does not (Busquets-Garcia et al. 2011). The relation between AEA concentrations and CB_1_ receptor density is out of phase and dependent on time of day. In the resting phase, a high CB_1_ receptor density together with low AEA concentrations is found in the hippocampus while this pattern is reversed when the animals are awake and active (Vaughn et al. 2010). Together, these results suggest that it is relevant to test memory during the morning, when AEA concentrations are high in humans, in addition to other times of the day, when AEA concentrations are lower, and assess the expression of the CB_1_ receptor, in a placebo-controlled MDMA study in order to know whether diurnal variations in AEA and CB_1_ receptor influence or change the MDMA-induced memory deficit.

Research into the role of eCBs in the MDMA-induced memory deficit in humans is limited by the absence of approved CB_1_ antagonists. Rimonabant, a CB_1_ receptor antagonist/inverse agonist, which was approved for weight control, was withdrawn from the market, in addition to a suspension of its development, after some adverse clinical psychiatric side effects (Janero and Makriyannis 2009). Another approach to this problem is to administer THC, a partial CB_1_ and CB_2_ agonist, or a fatty acid amide hydroxylase (FAAH) inhibitor, an enzyme involved in the catabolism of AEA, and MDMA in combination, since both combinations suggestible lead to an increase in endocannabinoid concentrations (Gobbi et al. 2005; Pertwee 1997). In animal research, a synergistic disruptive effect of the THC-MDMA combination was shown on memory (Young et al. 2005). One human study combining THC with MDMA did not show a change in the drug-induced memory effect of the single drugs on an N-back task (working memory). Interestingly, the subjective effects (e.g., drug strength, feeling high) did increase after the combined administration (Dumont et al. 2011). Interestingly, animal research has shown that order is important in these effects. While dopamine concentrations decreased when MDMA was given prior to THC, this change was not present when THC was given before MDMA (Robledo et al. 2007). In the study of Dumont et al. (2011), the first dose of THC (4 mg) was given concurrently with MDMA (100 mg), while the two subsequent doses of each 6 mg followed MDMA administration by 90 and 180 min. Future research could compare the effects of pre- and post-dosing MDMA-treated participants with THC to explore the order effects of the different treatments and reveal whether the effect on eCB concentrations relates to substance-induced memory impairment.

Although ketanserin exerted effects on the behavioral and biological level in the present study, inducing a response speed reduction in the word recognition task and causing an increase in AEA concentrations, it did not counteract the MDMA-induced memory impairment (immediate recall) which is in contrast to a previous study conducted by our group (van Wel et al. 2011). Findings of a previous study using the same dose (40 mg) of ketanserin suggest that this dose is sufficient to block the 5-HT_2_ receptor and potential subjective and/or behavioral effects caused by a serotonergic substance (Vollenweider et al. 1998). However, van Wel et al. (2011) used a 50-mg dose and showed a blockade of the MDMA-induced memory impairment. This 22% difference in dose resulted in ketanserin plasma concentrations that were 1.5 times lower than in the previous study. Scrutinizing behavioral data of both studies, it became apparent that memory performance during placebo (“baseline”) was comparable though the MDMA-induced impairment, relative to placebo, was larger in the study of van Wel et al. (2011). Performance in the latter study decreased with 29% compared to a 16% decrease in the present study. This apparent smaller decrease in memory performance after MDMA administration in the present study could potentially be attributed to the MDMA concentrations that were 20% lower. Previously, it was shown that MDMA-induced memory failures correlated positively with MDMA blood concentrations (Ramaekers et al. 2009). When MDMA was combined with ketanserin, the MDMA-induced decrease in memory performance was reduced with 15% while this was only 8% in the present study. This smaller “gain” in memory performance could then perhaps be attributed to the lower availability of ketanserin and hence a potentially smaller blockade of the 5-HT_2_ receptors. While behavioral and biological data showed some effects of ketanserin, proving that it had effects on the central level, these low concentrations in blood were possibly not strong enough to counteract the MDMA-induced memory impairment.

To conclude, current findings suggest that peripheral endocannabinoids are not related to verbal memory impairment during MDMA intoxication.
